# Hyperseries in the non-Archimedean ring of Colombeau generalized numbers

**DOI:** 10.1007/s00605-021-01647-0

**Published:** 2021-11-28

**Authors:** Diksha Tiwari, Paolo Giordano

**Affiliations:** University of Vienna, Vienna, Austria

**Keywords:** Colombeau generalized numbers, Non-archimedean rings, Generalized functions, 46F-XX, 46F30, 26E30

## Abstract

This article is the natural continuation of the paper: Mukhammadiev et al. *Supremum, infimum and hyperlimits of Colombeau generalized numbers* in this journal. Since the ring  of Robinson-Colombeau is non-Archimedean and Cauchy complete, a classical series $\sum _{n=0}^{+\infty }a_{n}$ of generalized numbers  is convergent *if* and only if $a_{n}\rightarrow 0$ in the sharp topology. Therefore, this property does not permit us to generalize several classical results, mainly in the study of analytic generalized functions (as well as, e.g., in the study of sigma-additivity in integration of generalized functions). Introducing the notion of hyperseries, we solve this problem recovering classical examples of analytic functions as well as several classical results.

## Introduction

In this article, the study of supremum, infimum and hyperlimits of Colombeau generalized numbers (CGN) we carried out in [13] is applied to the introduction of a corresponding notion of hyperseries. In [13], we recalled that $(x_{n})_{n\in \mathbb {N}}\in {\widetilde{\mathbb {R}}}^{\mathbb {N}}$ is a Cauchy sequence if and only if $\lim _{n\rightarrow +\infty }\left| x_{n+1}-x_{n}\right| =0$ (in the sharp topology). As a consequence, a series of CGN

1.1 $$\begin{aligned} \sum _{n\in \mathbb {N}}a_{n}\text { converges}\ \iff \ a_{n}\rightarrow 0\text { (in the sharp topology)}. \end{aligned}$$

Once again, this is a well-known property of every ultrametric space, cf., e.g., [11]. The point of view of the present work is that in a non-Archimedean ring such as , the notion of hyperseries , i.e. where we sum over the set of hyperfinite natural numbers , yields results which are more closely related to the classical ones, e.g. in studying analytic functions, sigma additivity and limit theorems for integral calculus, or in possible generalization of the Cauchy-Kowalevski theorem to generalized smooth functions (GSF; see e.g. [7]).

Considering the theory of analytic CGF as developed in [17] for the real case and in [20] for the complex one, it is worth to mention that several properties have been proved in both cases: closure with respect to composition, integration over homotopic paths, Cauchy integral theorem, existence of analytic representatives $u_{\varepsilon }$, a real analytic CGF is identically zero if it is zero on a set of positive Lebesgue measure, etc.  (cf. [14, 17, 20] and references therein). On the other hand, even if in [20] it is also proved that each complex analytic CGF can be written as a Taylor series, necessarily this result holds only in an infinitesimal neighborhood of each point. The impossibility to extend this property to a finite neighborhood is due to (1.1) and is hence closely related to the approach we follow in the present article.

We refer to [13] for notions such as the ring of Robinson-Colombeau, subpoints, hypernatural numbers, supremum, infimum and hyperlimits. See also [12] for a more general approach to asymptotic gauges. In the present paper, we focus only on examples and properties related to hyperseries, postponing those about analytic functions, integral calculus and the Cauchy-Kowalevski theorem to subsequent works. Once again, the ideas presented in the present article, which needs only [13] as prior knowledge, can surely be useful to explore similar ideas in other non-Archimedean Cauchy complete settings, such as [2, 3, 11, 19].

## Hyperseries and their basic properties

### Definition of hyperfinite sums and hyperseries

In order to define these notions, the main idea, like in the Archimedean case $\mathbb {R}$, is to reduce the notion of hyperseries to that of hyperlimit. Since to take an hyperlimit we have to consider two gauges $\rho$, $\sigma$, it is hence natural to consider the same assumption for hyperseries. However, in a non-Archimedean setting, the aforesaid main idea implies that the main problem is not in defining hyperseries itself, but already in defining what an hyperfinite sum is. In fact, if  is a family of generalized numbers of  indexed in , it is not even clear what $\sum _{i=0}^{4}a_{i}$, means because there are infinite  such that $0\le i\le 4$. In general, in a sum of the form $\sum _{i=0}^{N}a_{i}$ with , the number of addends depends on how small is the gauge $\sigma$ and hence how large can be $N\le \hbox {d}^{-R}$ (for some $R\in \mathbb {N}$). The problem is simplified if we consider only *ordinary* sequences $(a_{n})_{n\in \mathbb {N}}$ of CGN in  and an $\varepsilon$-wise definition of hyperfinite sum. In order to accomplish this goal, we first need to extend the sequence2.1of partial sums with summands , $n\in \mathbb {N}$, to the entire set  of hyperfinite numbers. This problem is not so easy to solve: in fact, the sequence of representatives of zero: $a_{n\varepsilon }=0$ if $\varepsilon \le \frac{1}{n}$ and $a_{n\varepsilon }=(1-\varepsilon )^{-n}$ otherwise, where $n\in \mathbb {N}_{>0}$, satisfies

2.2 $$\begin{aligned} \sum _{n=0}^{N_{\varepsilon }}a_{n\varepsilon }=\sum _{n=\left\lceil \frac{1}{\varepsilon }\right\rceil }^{N_{\varepsilon }}\left( \frac{1}{1-\varepsilon }\right) ^{n}\quad \forall \varepsilon \in (0,1)_{\mathbb {R}}, \end{aligned}$$

but if $N_{\varepsilon }\rightarrow +\infty$ this sum diverges to $+\infty$ because $\frac{1}{1-\varepsilon }>1$; moreover, for suitable $N_{\varepsilon }$, the net in (2.2) is of the order of $\left( \frac{1}{1-\varepsilon }\right) ^{N_{\varepsilon }}$, which in general is not $\rho$-moderate. To solve this first problem, we have at least two possibilities: the first one is to consider a Robinson-Colombeau ring defined by the index set $\mathbb {N}\times I$ and ordered by $(n,\varepsilon )\le (m,e)$ if and only if $\varepsilon \le e$. In this solution, moderate representatives are nets $(a_{n\varepsilon })_{n,\varepsilon }\in \mathbb {R}^{\mathbb {N}\times I}$ satisfying the uniformly moderate condition

2.3 $$\begin{aligned} \exists Q\in \mathbb {N}\,\forall ^{0}\varepsilon \,\forall n\in \mathbb {N}:\ |a_{n\varepsilon }|\le \rho _{\varepsilon }^{-Q}. \end{aligned}$$

Negligible nets are $(a_{n\varepsilon })_{n,\varepsilon }\in \mathbb {R}^{\mathbb {N}\times I}$ such that

2.4 $$\begin{aligned} \forall q\in \mathbb {N}\,\forall ^{0}\varepsilon \,\forall n\in \mathbb {N}:\ |a_{n\varepsilon }|\le \rho _{\varepsilon }^{q}. \end{aligned}$$

Note that, with respect to the aforementioned directed order relation, for any property $\mathcal {P}$, we have

$$\begin{aligned} \left( \exists (n_{0},\varepsilon _{0})\in \mathbb {N}\times I\,\forall (n,\varepsilon )\le (n_{0},\varepsilon _{0}):\ \mathcal {P}\left\{ n,\varepsilon \right\} \right) \ \iff \ \forall ^{0}\varepsilon \,\forall n\in \mathbb {N}:\ \mathcal {P}\left\{ n,\varepsilon \right\} . \end{aligned}$$

The main problem with this solution is that it works to define hyperfinite sums only if

2.5 $$\begin{aligned} \exists Q_{\sigma ,\rho }\in \mathbb {N}\,\forall ^{0}\varepsilon :\ \sigma _{\varepsilon }\ge \rho _{\varepsilon }^{Q_{\sigma ,\rho }}. \end{aligned}$$

Assume, indeed, that (2.3) holds for all $\varepsilon \le \varepsilon _{0}$, so that if , $N_{\varepsilon }\in \mathbb {N}$, we have

2.6 $$\begin{aligned} \left| \sum _{n=0}^{N_{\varepsilon }}a_{n\varepsilon }\right| \le N_{\varepsilon }\cdot \rho _{\varepsilon }^{-Q}\le {{\sigma }}_{\varepsilon }^{-R}\cdot \rho _{\varepsilon }^{-Q}\le \rho _{\varepsilon }^{-RQ_{\sigma ,\rho }-Q}, \end{aligned}$$

where we assumed that $N_{\varepsilon }\le \sigma _{\varepsilon }^{-R}$ for these $\varepsilon \le \varepsilon _{0}$. This is intuitively natural since the Robinson-Colombeau ring defined by the aforementioned order relation depends only on the gauge $\rho$, whereas the moderateness in (2.6) depends on the product $N_{\varepsilon }\cdot \rho _{\varepsilon }^{-Q}\le \sigma _{\varepsilon }^{-R}\cdot \rho _{\varepsilon }^{-Q}$ between how many addends we are taking (that depends on $\sigma$) and the uniform moderateness property (2.3).

This first solution has three drawbacks: The first one, as explained above, is that in this ring we can consider hyperfinite sum only if the relation (2.5) between the two considered gauges holds. The second one is that we cannot consider divergent hyperseries such as e.g.  because $n\le \rho _{\varepsilon }^{-Q}$ do not hold for $\varepsilon$ small and uniformly for all $n\in \mathbb {N}$. The third one is that we would like to apply [13, Thm. 28] to prove the convergence of hyperseries by starting from the corresponding converging series of $\varepsilon$-representatives; however, [13, Thm. 28] do not allow us to get the limitation (2.5) (and later, we will see that in general the limitation (2.5) is impossible to achieve: see just after the proof of Theorem 12).

The second possibility to extend (2.1) to , a possibility that depends on two gauges but works for any $\sigma$ and $\rho$, is to say that hyperseries can be computed only for representatives $(a_{n\varepsilon })_{n\varepsilon }$ which are *moderate over hypersums*, i.e. to ask2.7For example, if , then $\left( k_{\varepsilon }^{n}\right) _{n,\varepsilon }$ is moderate over hypersums (see also Example 8 below); but also the aforementioned $a_{n\varepsilon }=n$ for all $n\in \mathbb {N}$ and $\varepsilon \in I=(0,1]_{\mathbb {R}}$ is clearly of the same type if $\mathbb {R}_{\sigma }\subseteq \mathbb {R}_{\rho }$.

However, if $a_{n}=[a_{n\varepsilon }]=[\bar{a}_{n\varepsilon }]$ for all $n\in \mathbb {N}$ are two sequences which are moderate over hypersums, does the equality $\left[ \sum _{n=0}^{\mathrm{ni}{(N)}_{\varepsilon }}a_{n\varepsilon }\right] =\left[ \sum _{n=0}^{\mathrm{ni}{(N)}_{\varepsilon }}\bar{a}_{n\varepsilon }\right]$ hold? The answer is negative: let $a_{n\varepsilon }{:=}0$ if $\varepsilon <\frac{1}{n+1}$ and $a_{n\varepsilon }{:=}1$ otherwise, then $[a_{n\varepsilon }]=0$ are representatives of zero, but the corresponding series is not zero:2.8Note that both examples (2.2) and (2.8) show that in dealing with hypersums, also the values $a_{n\varepsilon }$ for “$\varepsilon$ large” may play a role. We can then proceed like for (2.7) by saying that $(a_{n\varepsilon })_{n,\varepsilon }\sim _{\sigma \rho }(\bar{a}_{n\varepsilon })_{n,\varepsilon }$ if2.9The idea of this second solution is hence to consider hyperseries only for representatives which are moderate over hypersums modulo the equivalence relation (2.9).

In the following definition, we will consider both solutions:

#### Definition 1

The quotient set  of the set $\left( \mathbb {R}^{\mathbb {N}\times I}\right) _{\sigma \rho }$ of nets which are $\sigma$, $\rho$-*moderate over hypersums*by $\sigma$, $\rho$-*negligible nets*is called the *space of sequences for hyperseries*. Nets of $\mathbb {R}^{\mathbb {N}\times I}$ are denoted as $(a_{n\varepsilon })_{n,\varepsilon }$ or simply as $(a_{n\varepsilon })$; equivalence classes of  are denoted as .

The ring of Robinson-Colombeau defined by the index set $\mathbb {N}\times I$ ordered by

2.10 $$\begin{aligned} (n,\varepsilon )\le (m,e)\ :\iff \varepsilon \le e, \end{aligned}$$

is denoted as . We recall that  is the quotient space of moderate nets $(a_{n\varepsilon })_{n,\varepsilon }$ (i.e. (2.3) holds) modulo negligible nets (i.e. (2.4) holds) with respect to the directed order relation (2.10).

The letter ’u’ in  recalls that in this case we are considering *uniformly *moderate (and *uniformly *negligible) sequences. The letter ’s’ in  recalls that this is the space of *sequences *for hyperseries. We recall that  is a ring with respect to pointwise multiplication $(a_{n})_{n}\cdot (b_{n})_{n}=\left[ \left( a_{n\varepsilon }\cdot b_{n\varepsilon }\right) _{n\varepsilon }\right]$. When we want to distinguish equivalence classes in these two quotient sets, we use the notationsNote explicitly that, on the contrary with respect to , the ring  depends on only one gauge $\rho$.

We already observed the importance to consider suitable relations between the two gauges $\sigma$ and $\rho$:

#### Definition 2

Let $\sigma$, $\rho$ be two gauges, then we define

$$\begin{aligned} \sigma&\ge \rho ^{*}\ :\iff \ \exists Q_{\sigma ,\rho }\in \mathbb {N}\,\forall ^{0}\varepsilon :\ \sigma _{\varepsilon }\ge \rho _{\varepsilon }^{Q_{\sigma ,\rho }}.\\ \sigma&\le \rho ^{*}\ :\iff \ \exists Q_{\sigma ,\rho }\in \mathbb {N}_{>0}\,\forall ^{0}\varepsilon :\ \sigma _{\varepsilon }\le \rho _{\varepsilon }^{Q_{\sigma ,\rho }}. \end{aligned}$$

(Note that if $\sigma \ge \rho ^{*}$, then $Q_{\sigma ,\rho }>0$ since $\sigma _{\varepsilon }\rightarrow 0$).

It is easy to prove that $(-)\le (-)^{*}$ is a reflexive, transitive and antisymmetric relation, in the sense that $\sigma \le \rho ^{*}$ and $\rho \le \sigma ^{*}$ imply $\sigma _{\varepsilon }=\rho _{\varepsilon }$ for $\varepsilon$ small, whereas $(-)\ge (-)^{*}$ is only a partial order. Clearly, $\sigma \ge \rho ^{*}$ is equivalent to the inclusion of $\sigma$-moderate nets $\mathbb {R}_{\sigma }\subseteq \mathbb {R}_{\rho }$, whereas $\sigma \le \rho ^{*}$ is equivalent to $\mathbb {R}_{\sigma }\supseteq \mathbb {R}_{\rho }$.

It is well-known that there is no natural product between two ordinary series in $\mathbb {R}$ and involving summations with only one index set in $\mathbb {N}$ (see e.g. [4] and Sect. 4 below). This is the main motivation to consider only a structure of -module on  and, later, the natural Cauchy product between hyperseries:

#### Theorem 3

 is a quotient -module.

#### Proof

The closure of the set of $\sigma$, $\rho$-moderate nets over hypersums, i.e.  with respect to pointwise sum $(a_{n\varepsilon }+b_{n\varepsilon })$ and product $(r_{\varepsilon })\cdot (a_{n,\varepsilon })=(r_{\varepsilon }\cdot a_{n,\varepsilon })$ by $(r_{\varepsilon })\in \mathbb {R}_{\rho }$ follows from similar properties of the ring $\mathbb {R}_{\rho }$. Similarly to the case of , we can finally prove that the equivalence relation (2.9) is a congruence with respect to these operations. $\square$

We now prove that if , then hyperfinite sums are well-defined:

#### Theorem 4

Let  and let $\sigma$, $\rho$ be two arbitrary gauges, then the mapis well-defined.

#### Proof

$\rho$-moderateness directly follows from ; in fact:

2.11 $$\begin{aligned} \left| \sum _{n=\mathrm{ni}{(N)}_{\varepsilon }}^{\mathrm{ni}{(M)}_{\varepsilon }}a_{n\varepsilon }\right| =\left| \sum _{n=0}^{\mathrm{ni}{(M)}_{\varepsilon }}a_{n\varepsilon }-\sum _{n=0}^{\mathrm{ni}{(N)}_{\varepsilon }-1}a_{n\varepsilon }\right| \le \rho _{\varepsilon }^{-Q_{1}}+\rho _{\varepsilon }^{-Q_{2}}. \end{aligned}$$

Now, assume that $(a_{n})_{n}=[\bar{a}_{n\varepsilon }]$ is another representative. For $q\in \mathbb {N}$, condition (2.9) yields

$$\begin{aligned} \left| \sum _{n=\mathrm{ni}{(N)}_{\varepsilon }}^{\mathrm{ni}{(M)}_{\varepsilon }}a_{n\varepsilon }-\sum _{n=\mathrm{ni}{(N)}_{\varepsilon }}^{\mathrm{ni}{(M)}_{\varepsilon }}\bar{a}_{n\varepsilon }\right|&\le \rho _{\varepsilon }^{q} \end{aligned}$$

for $$\varepsilon $$ small. $$\square $$

We can finally define hyperfinite sums and hyperseries.

#### Definition 5

Let  and let $\sigma$ and $\rho$ be two arbitrary gauges then the term2.12is called $\sigma$, $\rho$-*hypersum* of $(a_{n})_{n}$ (*hypersum* for brevity).

Moreover, we say that *s* *is the* $\rho$-*sum of hyperseries with terms* $(a_{n})_{n\in \mathbb {N}}$* as*  if *s* is the hyperlimit of the hypersequence . In this case, we writeIn case the use of the gauge $\rho$ is clear from the context, we simply say that *s* *is the sum of the hyperseries with terms* $(a_{n})_{n\in \mathbb {N}}$ *as* .

As usual, we also say that the hyperseries  is *convergent* ifWhereas, we say that a hyperseries  *does not converge* if  does not exist in . More specifically, if  ($-\infty$), we say that  *diverges to* $+\infty$ ($-\infty$).

For the sake of brevity, when dealing with hyperseries or with hypersums, we always *implicitly assume* that $\sigma$, $\rho$ are two gauges and that .

#### Remark 6

(i) Note that we are using an abuse of notations, since the term $\sum _{n=N}^{M}a_{n}$ actually depends on the two considered gauges $\rho$, $\sigma$.
(ii) Explicitly,  means 2.13
(iii) Also note that if *N*, $M\in \mathbb {N}$, then  is the usual finite sum. This is related to our motivating discussion about the definition of hyperfinite series at the beginning of Sect. 2.

### Relations between  and 

A first consequence of the condition $\sigma \ge \rho ^{*}$ is that if the net $(a_{n\varepsilon })$ is uniformly moderate, then it is also moderate over hypersums. Similarly, we can argue for the equality, so that we have a natural map :

#### Lemma 7

We have the following properties:

(i) If , with $N_{\varepsilon }\in \mathbb {N}$, and , then  is well defined. That is, any sequence  can be extended from $\mathbb {N}$ to the entire set  of hyperfinite numbers.
  Moreover, if we assume that $\sigma \ge \rho ^{*}$, then we have:
(ii) Both  and  via the embedding $[x_{\varepsilon }]\mapsto [(x_{\varepsilon })_{n,\varepsilon }]$.
(iii) The mapping  is a well-defined -linear map.
(iv) Let us define a hypersum operator  for all *M*, . Then . Therefore, the character (convergent or divergent) of the corresponding hyperseries is identical in the two spaces  and .

#### Proof

(i) In fact, if $(N_{\varepsilon })\in \mathbb {N}_{\sigma }$, then from  we get the existence of $Q_{i}\in \mathbb {N}$ such that
  $$\begin{aligned} \left| a_{N_{\varepsilon },\varepsilon }\right| =\left| \sum _{n=0}^{N_{\varepsilon }}a_{n\varepsilon }-\sum _{n=0}^{N_{\varepsilon }-1}a_{n\varepsilon }\right| \le \rho _{\varepsilon }^{-Q_{1}}+\rho _{\varepsilon }^{-Q_{2}}. \end{aligned}$$
  Finally, if , then directly from (2.9) with $M=N$ we get $[(a_{\mathrm{ni}{(N)}_{\varepsilon },\varepsilon })_{n,\varepsilon }]=[(\bar{a}_{\mathrm{ni}{(N)}_{\varepsilon },\varepsilon })_{n,\varepsilon }]$ (note that it is to have this result that we defined (2.9) using $\sum _{\mathrm{ni}{(N)}_{\varepsilon }}^{\mathrm{ni}{(M)}_{\varepsilon }}$ instead of $\sum _{n=0}^{\mathrm{ni}{(N)}_{\varepsilon }}$ like in (2.7)). Therefore,  is well-defined. In particular, this applies with $N=n\in \mathbb {N}$, so that any equivalence class  also defines an ordinary sequence $(a_{n})_{n\in \mathbb {N}}=\left( \left[ a_{n\varepsilon }\right] \right) _{n\in \mathbb {N}}$ of . On the other hand, let us explicitly note that if $(a_{n})_{n\in \mathbb {N}}=(\bar{a}_{n})_{n\in \mathbb {N}}$, i.e. if $a_{n}=\bar{a}_{n}$ for all $n\in \mathbb {N}$, then not necessarily (2.9) holds, i.e. we can have $(a_{n})_{n}\ne (\bar{a}_{n})_{n}$ as elements of the quotient module .
Claim (ii) is left to the reader.
(iii) Assume that inequality in (2.3) holds for $\varepsilon \le \varepsilon _{0}$, i.e.
  2.14 $$\begin{aligned} \forall \varepsilon \le \varepsilon _{0}\,\forall n\in \mathbb {N}:\ \left| a_{n\varepsilon }\right| \le \rho _{\varepsilon }^{-Q}. \end{aligned}$$
  Without loss of generality, we can also assume that $\mathrm{ni}{(N)}_{\varepsilon }+1\le \sigma _{\varepsilon }^{-R}$ and $\sigma _{\varepsilon }\ge \rho _{\varepsilon }^{-Q_{\sigma ,\rho }}$. Then, for each $\varepsilon \le \varepsilon _{0}$, using (2.14) we have $\left| \sum _{n=0}^{\mathrm{ni}{(N)}_{\varepsilon }}a_{n\varepsilon }\right| \le \left( \mathrm{ni}{(N)}_{\varepsilon }+1\right) \cdot \rho _{\varepsilon }^{-Q}\le \sigma _{\varepsilon }^{-R}\cdot \rho _{\varepsilon }^{-Q}\le \rho _{\varepsilon }^{-R\cdot Q_{\sigma ,\rho }-Q}$. Moreover, if $\left[ a_{n\varepsilon }\right] _{\text {u}}=0$ then, for all $q\in \mathbb {N}$
  $$\begin{aligned} \left| \sum _{n=0}^{\mathrm{ni}{(N)}_{\varepsilon }}a_{n\varepsilon }\right| \le \left( \mathrm{ni}{(N)}_{\varepsilon }+1\right) \cdot \rho _{\varepsilon }^{q} \end{aligned}$$
  for $\varepsilon$ small. Since $\mathrm{ni}{(N)}_{\varepsilon }$ is $\sigma$-moderate and $\sigma \ge \rho ^{*}$, this proves that the linear map $\lambda$ is well-defined.
(iv) This follows directly from Definition 5. $\square$

It remains an open problem the study of injectivity of the map $\lambda$.

We could say that $\sigma \ge \rho ^{*}$ and  are sufficient conditions to get  and hence to start talking about hyperfinite sums $\sum _{n=N}^{M}a_{n}$ and hyperseries . On the other hand, if $\sigma \ge \rho ^{*}$ is false, we can still consider the space  and hence talk about hyperseries, but the corresponding space  lacks elements such as $(1)_{n}$ because of the subsequent Lemma 10. As we will see in the following example 8 (3) and (6), the space  still contains sequences corresponding to interesting converging hyperseries.

The following examples of convergent hyperseries justify our definition of hyperseries by recovering classical examples such such geometric and exponential hyperseries. We recall that this is not possible using classical series in a non-Archimedean setting.

#### Example 8

1. Let , where $N_{\varepsilon }\in \mathbb {N}$ for all $\varepsilon$, then . We recall that $a_{N}=\left[ a_{N_{\varepsilon },\varepsilon }\right]$ is the extension of  to  (see (i) of Lemma 7).
2. For all , $0<k<1$, we have (note that $\sigma =\rho$) 2.15 We first note that $k^{n}\le 1$, so that . Now,  But $k^{N+1}<\hbox {d}^{q}$ if and only if $(N+1)\log k<q\log \hbox {d}$. Since $0<k<1$, we have $\log k<0$ and we obtain $N>q\frac{\log \hbox {d}}{\log k}-1$. It suffices to take $M_{\varepsilon }{:=}\text {int}\left( q\frac{\log \rho _{\varepsilon }}{\log k_{\varepsilon }}\right)$ in the definition of hyperseries.
3. More generally, if , $0<k<1$, we can evaluate  This shows that  for all gauges $\sigma$. If we assume $\sigma \le \rho ^{*}$ then $M_{\varepsilon }{:=}\text {int}\left( q\frac{\log \rho _{\varepsilon }}{\log k_{\varepsilon }}\right) \in \mathbb {N}_{\sigma }$ and hence, proceeding as above, we can prove that .
4. Let  be such that $k>_{\mathrm{s}}1$ (see [13] for the relations $>_{\mathrm{s}}$ and $=_{\mathrm{s}}$ and, more generally, for the language of subpoints), then the hyperseries  is not convergent. In fact, by contradiction, in the opposite case we would have $\sum _{n=0}^{N}k^{n}\in (l-1,l+1)$ for some  for all *N* sufficiently large, but this is impossible because for all fixed  and for *N* sufficiently large,  because .
5. For all  finite, we have . We have $|x|<M\in \mathbb {R}_{>0}$ because *x* is finite, and hence $\left| x_{\varepsilon }\right| \le M$ for all $\varepsilon \le \varepsilon _{0}$. Thereby $\frac{x_{\varepsilon }^{n}}{n!}\le \frac{\left| x_{\varepsilon }\right| ^{n}}{n!}\le e^{M}$ for all $n\in \mathbb {N}$ and thus . For all , $N_{\varepsilon }\in \mathbb {N}$, and all $\varepsilon$, we have
  2.16 $$\begin{aligned} \sum _{n=0}^{N_{\varepsilon }}\frac{x_{\varepsilon }^{n}}{n!}=e^{x_{\varepsilon }}-\sum _{n=N_{\varepsilon }+1}^{+\infty }\frac{x_{\varepsilon }^{n}}{n!}. \end{aligned}$$
  Now, take  such that $\frac{M}{N+1}<\frac{1}{2}$, so that we can assume $N_{\varepsilon }+1>2M$ for all $\varepsilon$. We have $\left| \sum _{n=N_{\varepsilon }+1}^{+\infty }\frac{x_{\varepsilon }^{n}}{n!}\right| \le \sum _{n>N_{\varepsilon }}\frac{M^{n}}{n!}$, and for all $n\ge N_{\varepsilon }$ we have (by induction)
  $$\begin{aligned} \frac{M^{n+1}}{(n+1)!}<\frac{1}{2^{n+1}}. \end{aligned}$$
  Therefore $\left| \sum _{n=N_{\varepsilon }+1}^{+\infty }\frac{x_{\varepsilon }^{n}}{n!}\right| \le \sum _{n>N_{\varepsilon }}\frac{1}{2^{n}}$ and hence  by (2.15). This and (2.16) yields the conclusion.
6. In the same assumptions of the previous example, we have $\left[ \left| \sum _{n=0}^{N_{\varepsilon }}\frac{x_{\varepsilon }^{n}}{n!}\right| \right] \le e^{M}$ and hence  for all gauges $\sigma$. If $\sigma \le \rho ^{*}$, proceeding as above, we can prove that .

Finally, the following lemma shows that a sharply bounded sequence of  always defines a sequence for hyperseries, i.e. an element of .

#### Lemma 9

Let $(a_{n})_{n\in \mathbb {N}}$ be a sequence of . If $(a_{n})_{n\in \mathbb {N}}$ is sharply bounded:2.17then there exists a sequence $(a_{n\varepsilon })_{n\in \mathbb {N}}$ of $\mathbb {R}_{\rho }$ such that

(i) for all $$n\in \mathbb {N}$$;
(ii);
(iii) If .

#### Proof

Let $M=[M_{\varepsilon }]$ be any representative of the bound satisfying (2.17), so that $M_{\varepsilon }\le \rho _{\varepsilon }^{-Q}$ for $\varepsilon \le \varepsilon _{0}$ and for some $Q\in \mathbb {N}$. From (2.17), for each $n\in \mathbb {N}$ we get the existence of a representative $a_{n}=[\bar{a}_{n\varepsilon }]$ such that $|\bar{a}_{n\varepsilon }|\le M_{\varepsilon }$ for $\varepsilon \le \varepsilon _{0n}\le \varepsilon _{0}$. It suffices to define $a_{n\varepsilon }{:=}\bar{a}_{n\varepsilon }$ if $\varepsilon \le \varepsilon _{0n}$ and $a_{n\varepsilon }{:=}M_{\varepsilon }$ otherwise to have $\forall \varepsilon \le \varepsilon _{0}\,\forall n\in \mathbb {N}:\ \left| a_{n\varepsilon }\right| \le \rho _{\varepsilon }^{-Q}$, so that . If $\sigma \ge \rho ^{*}$, we can then apply Lemma 7. $\square$

However, let us note that, generally speaking, changing representatives of $a_{n}$ as in the previous proof, we also get a different value of the corresponding hyperseries, as proved by example (2.8).

### Divergent hyperseries

By analyzing when the constant net (1) is moderate over hypersums, we discover the relation (2.5) between the gauges $\sigma$ and $\rho$:

#### Lemma 10

The constant net $(1)\in (\mathbb {R}^{\mathbb {N}\times I})_{\sigma \rho }$, i.e. it is $\sigma$, $\rho$-moderate over hypersums, if and only if $\sigma \ge \rho ^{*}$.

#### Proof

If $(1)\in (\mathbb {R}^{\mathbb {N}\times I})_{\sigma \rho }$, we set $N_{\varepsilon }{:=}\text {int}(\sigma _{\varepsilon }^{-1})+1$, so that we get $\sigma _{\varepsilon }^{-1}\le \sum _{n=0}^{N_{\varepsilon }}1=N_{\varepsilon }\le \rho _{\varepsilon }^{-Q_{\sigma ,\rho }}$ for some $Q_{\sigma ,\rho }\in \mathbb {N}$, i.e. $\sigma \ge \rho ^{*}$. Vice versa, if $\sigma _{\varepsilon }\ge \rho _{\varepsilon }^{Q_{\sigma ,\rho }}$ for all $\varepsilon \le \varepsilon _{0}$, then for those $\varepsilon$, we have $\left| \sum _{n=0}^{\mathrm{ni}{(N)}_{\varepsilon }}1\right| =\mathrm{ni}{(N)}_{\varepsilon }\le \sigma _{\varepsilon }^{-R}\le \rho _{\varepsilon }^{-R\cdot Q_{\sigma ,\rho }}$ for some $R\in \mathbb {N}$, because . $\square$

One could argue that we are mainly interested in converging hyperseries and hence it is not worth considering the constant net (1). On the other hand, we would like to argue in the following way: the hypersums  can be considered, but they do not converge because $1\not \rightarrow 0$. As we will see in Lemma 15, this argumentation is possible only if $\sigma \ge \rho ^{*}$ because of the previous Lemma 10.

Let $\sigma \ge \rho ^{*}$ and  be an infinite number. If , we can also think at $\sum _{n=0}^{\omega }a_{n}$ as another way to compute an infinite summation of the numbers $a_{n}$. In other words, the following examples can be considered as related to calculation of divergent series. They strongly motivate and clarify our definition of hyperfinite sum.

#### Example 11

Assuming $\sigma \ge \rho ^{*}$ and working with the module , we have:

(5).
(6) $$\sum _{n=1}^{\omega }n=\left[ \sum _{n=1}^{\mathrm{ni}{(\omega )}_{\varepsilon }}n_{\varepsilon }\right] =\left[ \frac{\mathrm{ni}{(\omega )}_{\varepsilon }(\mathrm{ni}{(\omega )}_{\varepsilon }+1)}{2}\right] =\frac{\omega (\omega +1)}{2}$$.
(7) $\sum _{n=1}^{\omega }(2n-1)=2\sum _{n=1}^{\omega }n-\sum _{n=1}^{\omega }1=\omega ^{2}$ because we know from Theorem 3 that  is an -module.
(8) $$\sum _{n=1}^{\omega }\left( a+(n-1)d\right) =\omega a+\frac{\omega ^{2}d}{2}-\frac{\omega d}{2}$$.
(9) Using $\varepsilon$-wise calculations, we also have $\sum _{n=0}^{\omega }(-1)^{n}=\frac{1}{2}(-1)^{\omega +1}+\frac{1}{2}$. Note that the final result is a finite generalized number of , but it does not converge for $\omega \rightarrow +\infty$, .
10) The net $(2^{n})_{n,\varepsilon }$ is not $\rho$-moderate over hypersums, in fact if $\omega =\left[ \text {int}(\rho _{\varepsilon }^{-1})\right]$, then $\sum _{n=0}^{\omega _{\varepsilon }}2^{n-1}=2^{\omega _{\varepsilon }}-1$, which is not $\rho$-moderate. Another possibility to consider the function $2^{\omega }$ is to take another gauge $\mu \le \rho$ and the subring of  defined by  where only here we have set . If we have
  2.18 $$\begin{aligned} \forall N\in \mathbb {N}\,\exists M\in \mathbb {N}:\ \hbox {d}^{-N}\le -M\log \hbox {d}, \end{aligned}$$
  then  is well defined. For example, if $\mu _{\varepsilon }{:=}\exp \left( -\rho _{\varepsilon }^{1/\varepsilon }\right)$, then $\mu \le \rho$ and (2.18) holds for $M=1$. Note that the natural ring morphism  is surjective but generally not injective. Now, if , then .
(11) Once again, proceeding by $\varepsilon$-wise calculations, we also have the binomial formula: For all *a*,  and , if , then
  $$\begin{aligned} \left( a+b\right) ^{n}=\sum _{k=0}^{n}\left( \begin{array}{c} n\\ k \end{array}\right) a^{k}b^{n-k} \end{aligned}$$
  where $$n!{:=}\left[ \text {ni }(n)_{\varepsilon }!\right] $$ and $$\left( \begin{array}{c} n\\ k \end{array}\right) {:=}\frac{n!}{k!\left( n-k\right) !}$$.

### $\varepsilon$-wise convergence and hyperseries

The following result allows us to obtain hyperseries by considering $\varepsilon$-wise convergence of its summands. Its proof is clearly very similar to that of [13, Theorem 28], but with a special attention to the condition  that we need beforehand to talk about hyperseries.

#### Theorem 12

Let  and $a_{n}=[a_{n\varepsilon }]$ for all $n\in \mathbb {N}$. Let $q_{\varepsilon }$, $M_{\varepsilon }\in \mathbb {N}_{>0}$ be such that $(q_{\varepsilon })\rightarrow +\infty$ as $\varepsilon \rightarrow 0^{+}$, and

2.19 $$\begin{aligned} \forall \varepsilon \,\forall N\ge M_{\varepsilon }:\ s_{\varepsilon }-\rho _{\varepsilon }^{q_{\varepsilon }}<\sum _{n=0}^{N}a_{n\varepsilon }<s_{\varepsilon }+\rho _{\varepsilon }^{q_{\varepsilon }} \end{aligned}$$

(note that this implies that the standard series $\sum _{n=0}^{+\infty }a_{n\varepsilon }$ converges to $s_{\varepsilon }$). Finally, let $\mu$ be another gauge. If , then setting $\sigma _{\varepsilon }{:=}\min (\mu _{\varepsilon },M_{\varepsilon }^{-1})$, we have:

(i) $\sigma \le \mu$ is a gauge (not necessarily a monotonic one);
(ii);
(iii);
(iv);
(v).

#### Proof

The net $\sigma _{\varepsilon }=\min (\mu _{\varepsilon },M_{\varepsilon }^{-1})\rightarrow 0^{+}$ as $\varepsilon \rightarrow 0^{+}$ because $\mu _{\varepsilon }\rightarrow 0^{+}$, i.e. it is a gauge (note that not necessarily $\sigma$ is non-decreasing, e.g. if $\lim _{\varepsilon \rightarrow \frac{1}{k}}M_{\varepsilon }=+\infty$ for all $k\in \mathbb {N}_{>0}$ and $M_{\varepsilon }\ge \mu _{\varepsilon }^{-1}$). We have  because our definition of $\sigma$ yields $M_{\varepsilon }\le \sigma _{\varepsilon }^{-1}$. Moreover, since $(q_{\varepsilon })\rightarrow +\infty$, for all $\varepsilon$ condition (2.19) yields $s_{\varepsilon }-1\le s_{\varepsilon }-\rho _{\varepsilon }^{q_{\varepsilon }}<\sum _{n=0}^{N}a_{n\varepsilon }<s_{\varepsilon }+\rho _{\varepsilon }^{q_{\varepsilon }}\le s_{\varepsilon }+1$ for all $N\ge M_{\varepsilon }$. For $N=M$ this gives , because we assumed that . If , then $\mathrm{ni}{(N)}_{\varepsilon }+M_{\varepsilon }\ge M_{\varepsilon }$ for all $\varepsilon$, and hence $s_{\varepsilon }-1<\sum _{n=0}^{M_{\varepsilon }-1}a_{n\varepsilon }+\sum _{n=M_{\varepsilon }}^{\mathrm{ni}{(N)}_{\varepsilon }+M_{\varepsilon }}a_{n\varepsilon }=s_{M\varepsilon }+\sum _{n=0}^{\mathrm{ni}{(N)}_{\varepsilon }}a_{n+M_{\varepsilon },\varepsilon }<s_{\varepsilon }+1$. This shows that , because $s_{M}$, , i.e. . Similarly, if $q\in \mathbb {N}$, then $q<q_{\varepsilon }$ for $\varepsilon$ sufficiently small and, proceeding as above, we can prove that $|\sum _{n=0}^{N}a_{n+M}-s-s_{M}|<\hbox {d}^{q}$. $\square$

Note that, if $(M_{\varepsilon })$ is not $\rho$-moderate and we set $\sigma _{\varepsilon }{:=}\min \left( \rho _{\varepsilon },M_{\varepsilon }^{-1}\right) \in (0,1]$, then

$$\begin{aligned} \forall Q\in \mathbb {N}\,\exists L\subseteq _{0}I\,\forall \varepsilon \in L:\ \sigma _{\varepsilon }^{-1}>\bar{M}_{\varepsilon }>\rho _{\varepsilon }^{-Q} \end{aligned}$$

and this shows that $\sigma \ge \rho ^{*}$, i.e. the fundamental condition to have moderateness of hyperfinite sums in the ring  (see (2.6)), *always necessarily does not hold*. On the other hand, if $(M_{\varepsilon })$ is $\rho$-moderate, then $\mathbb {R}_{\sigma }=\mathbb {R}_{\mu }$; in particular, we can simply take $\sigma =\mu =\rho$.

Assuming absolute convergence, we can obtain a stronger and clearer result:

#### Theorem 13

Let  and $a_{n}=[a_{n\varepsilon }]$ for all $n\in \mathbb {N}$. Assume that the standard series $\sum _{n=0}^{+\infty }a_{n\varepsilon }$ converges absolutely to $\bar{s}_{\varepsilon }$ and . Finally, let $\mu$ be another gauge and set $s_{\varepsilon }{:=}\sum _{n=0}^{+\infty }a_{n\varepsilon }$, then there exists a gauge $\sigma \le \mu$ such that:

(i);
(ii).

#### Proof

Set $q_{\varepsilon }{:=}\left\lceil \frac{1}{\varepsilon }\right\rceil$, so that the absolute convergence of $\sum _{n=0}^{+\infty }a_{n\varepsilon }$ yields

2.20 $$\begin{aligned} \forall \varepsilon \,\exists M_{\varepsilon }\in \mathbb {N}_{>0}\,\forall N\ge M_{\varepsilon }:\ \bar{s}_{\varepsilon }-\rho _{\varepsilon }^{q_{\varepsilon }}<\sum _{n=0}^{N}\left| a_{n\varepsilon }\right| <\bar{s}_{\varepsilon }+\rho _{\varepsilon }^{q_{\varepsilon }} \end{aligned}$$

and we can thereby apply the previous Theorem 12 obtaining $\bar{s}_{M}{:=}\left[ \sum _{n=0}^{M_{\varepsilon }-1}|a_{n\varepsilon }|\right]$, . Therefore, for an arbitrary , we have:and this shows that . Now, proceeding as above we can prove that . $\square$

### Basic properties of hyperfinite sums and hyperseries

We now study some basic properties of hyperfinite sums (2.12).

#### Lemma 14

Let $\sigma$, $\rho$ be arbitrary gauges, , and *M*, , then

2.21 $$\begin{aligned} \sum _{n=0}^{N+M}a_{n}-\sum _{n=0}^{N}a_{n}=\sum _{n=N+1}^{N+M}a_{n}. \end{aligned}$$

#### Proof

For simplicity, if $N=[N_{\varepsilon }]$, $M=[M_{\varepsilon }]$ with $N_{\varepsilon },M_{\varepsilon }\in \mathbb {N}$ for all $\varepsilon$, then  and

$$\begin{aligned} \sum _{n=0}^{N+M}a_{n}&=\left[ \sum _{n=0}^{N_{\varepsilon }+M_{\varepsilon }}a_{n\varepsilon }\right] \\&=\left[ \sum _{n=0}^{N_{\varepsilon }}a_{n\varepsilon }+\sum _{N_{\varepsilon }+1}^{N_{\varepsilon }+M_{\varepsilon }}a_{n\varepsilon }\right] \\&=\sum _{n=0}^{N}a_{n}+\sum _{n=N+1}^{N+M}a_{n}. \end{aligned}$$

$$\square $$

#### Lemma 15

If  is convergent and , thenTherefore, from (2.21), we also haveIn particular, .

#### Proof

Let . Directly from the definition of convergent hyperseries (2.13), we have2.22In particular, if , then also , which is our conclusion. $\square$

Directly from Lemma 14, we also have:

#### Corollary 16

Let  be a convergent hyperseries. Then adding a hyperfinite number of terms have no effect on the convergence of the hyperseries, that is

Mimicking the classical theory, we can also say that the hyperseries  is *Cesàro hypersummable* if  (this hyperlimit is clearly called *Cesàro hypersum*). For example, proceeding $\varepsilon$-wise, we have that the hyperseries  has Cesàro hypersum equal to $\frac{1}{2}$. Trivially generalizing the usual proof, we can show that if  converges to *s*, then it is also Cesàro hypersummable with the same hypersum *s*.

## Hyperseries convergence tests

### *p*-hyperseries

To deal with *p*-hyperseries, i.e. hyperseries of the formwe always assume that:

(i) is a *finite* generalized number such that $p>0$ or $p<0$; we recall that if *p* is an infinite number (at least on a subpoint), the operation $n^{p}$ is in general not well-defined since it leads to a non $\rho$-moderate net (see also Example 11.10)).
(ii) The gauges $\sigma$ and $\rho$ are chosen so that . A *sufficient* condition for this is that $\sigma \ge \rho ^{*}$, i.e. that $\mathbb {R}_{\sigma }\subseteq \mathbb {R}_{\rho }$. In fact, since *p* is finite, we have $\left( \sum _{n=1}^{N_{\varepsilon }}n^{-p_{\varepsilon }}\right) \le N_{\varepsilon }N_{\varepsilon }^{-p_{\varepsilon }}\in \mathbb {R}_{\sigma }\subseteq \mathbb {R}_{\rho }$ whenever $N_{\varepsilon }\in \mathbb {N}$, $\left( N_{\varepsilon }\right) \in \mathbb {N}_{\sigma }$.

Note explicitly that, from the assumption , we hence have3.1even if , and  is a different quotient ring with respect to  (e.g., in general we do *not* have ). In other words, it is not correct to say that the left hand side of (3.1) is the sum for  of terms , but instead it is a way to use $(N_{\varepsilon })\in \mathbb {N}_{\sigma }$ to compute a generalized number of .

If $p<0$, the general term $\frac{1}{n^{p}}\not \rightarrow 0$ because $n^{-p}\le \hbox {d}^{q}$ only if $n<\hbox {d}^{-q/p}$. Therefore, Lemma 15 yields that the *p*-series diverges.  We can hence consider only the case $p>0$. Let us note that Theorem 4, which is clearly based on our Definition 1 of , allows us to consider partial hypersums of the form $\sum _{n=1}^{N}\frac{1}{n^{p}}$ even if $p<0$. In other words, we can correctly argue that the *p*-hyperseries does not converge if $p<0$ not because the partial hypersums cannot be computed, but because the general term does not converge to zero. The situation would be completely different if we restrict our attention only to sequences $(a_{n})_{n}$ satisfying the (more natural) uniformly moderate condition (2.3). Similar remarks can be formulated for the calculus of divergent hyperseries in Example 8, 6)-10).

To prove the following results, we will follow some ideas of [10].

#### Theorem 17

Let  and let $S_{N}(p)$ be the *N*-th partial sum of the *p*-hyperseries, where , then

3.2 $$\begin{aligned} 1-\frac{1}{2^{p}}+\frac{2}{2^{p}}S_{N}(p)<S_{2N}(p)<1+\frac{2}{2^{p}}S_{N}(p). \end{aligned}$$

#### Proof

Let , with $N_{\varepsilon }\in \mathbb {N}_{\ge 1}$ for all $\varepsilon$, and $p=[p_{\varepsilon }]$, where $p_{\varepsilon }>0$ for all $\varepsilon$. Since

$$\begin{aligned} S_{2N}(p)&=\left[ \sum _{n=1}^{2N_{\varepsilon }}\frac{1}{n^{p_{\varepsilon }}}\right] =\left[ 1+\frac{1}{2^{p_{\varepsilon }}}+\frac{1}{3^{p_{\varepsilon }}}+\dots +\frac{1}{(2N_{\varepsilon }))^{p_{\varepsilon }}}\right] \\&=1+\left[ \frac{1}{2^{p_{\varepsilon }}}+\frac{1}{4^{p_{\varepsilon }}}+\dots +\frac{1}{((2N_{\varepsilon }))^{p_{\varepsilon }}}\right] \\ {}&\quad +\left[ \frac{1}{3^{p_{\varepsilon }}}+\frac{1}{5^{p_{\varepsilon }}}+\dots +\frac{1}{((2N_{\varepsilon }-1))^{p_{\varepsilon }}}\right] . \end{aligned}$$

But

3.3 $$\begin{aligned} 1+\left[ \frac{1}{2^{p_{\varepsilon }}}+\frac{1}{4^{p_{\varepsilon }}}+\dots +\frac{1}{((2N_{\varepsilon }))^{p_{\varepsilon }}}\right] =1+\frac{1}{2^{p}}S_{N}(p) \end{aligned}$$

and, since $p_{\varepsilon }>0$, we also have

3.4 $$\begin{aligned} \left[ \frac{1}{3^{p_{\varepsilon }}}+\frac{1}{5^{p_{\varepsilon }}}+\dots +\frac{1}{((2N_{\varepsilon }-1))^{p_{\varepsilon }}}\right]&>\left[ \frac{1}{4^{p_{\varepsilon }}}+\frac{1}{6^{p_{\varepsilon }}}+\dots +\frac{1}{((2N_{\varepsilon }))^{p_{\varepsilon }}}\right] \nonumber \\&=\frac{1}{2^{p}}S_{N}(p)-\frac{1}{2^{p}}. \end{aligned}$$

Summing (3.3) and (3.4) yields $S_{2N}(p)>1-\frac{1}{2^{p}}+\frac{2}{2^{p}}S_{N}(p)$. Finally, from (3.3) and

$$\begin{aligned} \left[ \frac{1}{3^{p_{\varepsilon }}}+\frac{1}{5^{p_{\varepsilon }}}+\dots +\frac{1}{((2N_{\varepsilon }-1))^{p_{\varepsilon }}}\right]&<\left[ \frac{1}{2^{p_{\varepsilon }}}+\frac{1}{4^{p_{\varepsilon }}}+\dots +\frac{1}{((2N_{\varepsilon }))^{p_{\varepsilon }}}\right] =\\&=\frac{1}{2^{p}}S_{N}(p) \end{aligned}$$

we get $$S_{2N}(p)<1+\frac{1}{2^{p}}S_{N}(p)+\frac{1}{2^{p}}S_{N}(p)$$. $$\square $$

#### Theorem 18

The *p*-hyperseries is divergent when $0<p\le _{\mathrm{s}}1$. When $p>1$, there exist some gauge $\tau \le \rho$ such that  and the *p*-series is convergent with respect to the gauges $\tau$, $\rho$ (to the usual real value $\zeta (p)$ if $p\in \mathbb {R}$), and in this case3.5

#### Proof

If $0<p\le _{\mathrm{s}}1$, there exist $J\subseteq _{0}I$ such that $p|_{J}\le 1$.Assume that the $p-$hyperseries is convergent and set . Taking $N\rightarrow \infty$ in the first inequality in (3.2), we have

$$\begin{aligned} 1-{{\frac{1}{2^{p}}+\frac{2}{2^{p}}S(p)}}=\frac{2^{p}-1}{2^{p}}+\frac{2}{2^{p}}S(p)\le S(p). \end{aligned}$$

Since for $\varepsilon \in J$ sufficiently small we have $0<p_{\varepsilon }<1$ for some $[p_{\varepsilon }]=p$, we obtain

$$\begin{aligned} 0<\frac{2^{p_{\varepsilon }}-1}{2^{p_{\varepsilon }}}\le \frac{2^{p_{\varepsilon }}-2}{2^{p_{\varepsilon }}}S(p)\le 0. \end{aligned}$$

This shows that the $p-$hyperseries is divergent when $p\le _{\mathrm{s}}1$. Now let $p>1$, so that we can assume $p_{\varepsilon }>1$ for all $\varepsilon$. From (3.2), we have

$$\begin{aligned} S_{N_{\varepsilon }}(p_{\varepsilon })<S_{2N_{\varepsilon }}(p_{\varepsilon })<1+\frac{2}{2^{p_{\varepsilon }}}S_{N_{\varepsilon }}(p_{\varepsilon }), \end{aligned}$$

where $$S_{N_{\varepsilon }}(p_{\varepsilon })=\sum _{n=1}^{N_{\varepsilon }}\frac{1}{n^{p_{\varepsilon }}}\in \mathbb {R}$$. Thereby

$$\begin{aligned}&0<\left( 1-\frac{2}{2^{p_{\varepsilon }}}\right) S_{N_{\varepsilon }}(p_{\varepsilon })<1\\&S_{N_{\varepsilon }}(p_{\varepsilon })<\frac{2^{p_{\varepsilon }}}{2^{p_{\varepsilon }}-2}. \end{aligned}$$

So $S_{N_{\varepsilon }}(p)$ is bounded and increasing, and applying Theorem 12, we get the existence of a gauge $\tau$ such that  and  converges to $\sum _{n=1}^{+\infty }\frac{1}{n^{p}}=\zeta (p)$. $\square$

Later, in Corollary 34 and using the integral test Thm. 33, we will prove the convergence of *p*-hyperseries for arbitrary gauges $\sigma$, $\rho$ (even if we will not get the estimates (3.5)).

### Absolute convergence

#### Theorem 19

If  converges then also  converges.

#### Proof

Cauchy criterion [13, Thm. 37], yieldsThe conclusion hence follows from the inequality

$$\begin{aligned} \left| \sum _{n=0}^{N_{1}}a_{n}-\sum _{n=0}^{N_{2}}a_{n}\right| <\left| \sum _{n=0}^{N_{1}}|a_{n}|-\sum _{n=0}^{N_{2}}|a_{n}|\right| , \end{aligned}$$

and from Cauchy criterion for hypersequences, i.e. [13, Thm. 37]. $\square$

### Direct and limit comparison tests

In the direct comparison test, we need to assume a relation of the form $a_{n}\le b_{n}$ for all $n\in \mathbb {N}$ between general terms of two hyperseries. If $a_{n}=[a_{n\varepsilon }]$,  are two representatives, then this inequality would yieldThe dependence of $\varepsilon _{0n}$ from $n\in \mathbb {N}$ does not allow to prove, e.g., that $\sum _{n=0}^{N}a_{n}\le \sum _{n=0}^{N}b_{n}$ (e.g. as in (2.8), we can consider $b_{n\varepsilon }{:=}0$ and $a_{n\varepsilon }{:=}0$ if $\varepsilon <\frac{1}{n+1}$ but $a_{n\varepsilon }{:=}1$ otherwise, then $a_{n}=[a_{n\varepsilon }]=0=b_{n}$, but $\sum _{n=0}^{N}a_{n}=N-\left[ \left\lceil \frac{1}{\varepsilon }\right\rceil \right] +2$).

Moreover, for convergence tests we also need to perform pointwise (in $n\in \mathbb {N}$) operations of the form $\left( \frac{a_{n}}{b_{n}}\right) _{n}$, $\left( \left| \frac{a_{n+k}}{a_{n}}\right| \right) _{n}$ or $\left( \left| a_{n}\right| ^{1/n}\right) _{n}$. This kind of pointwise operations can be easily considered if we restrict us to sequences  and we assume that $\sigma \ge \rho ^{*}$. Anyway, this is a particular sufficient condition, and we can more generally state some convergence tests using the more general space . For these reasons, we define

#### Definition 20

Let $(a_{n})_{n}$, , then we say that $(a_{n})_{n}\le (b_{n})_{n}$ if3.6Note explicitly that if $M<_{L}N$ on $L\subseteq _{0}I$, then $\sum _{n=N}^{M}a_{n}=_{L}\sum _{n=N}^{M}b_{n}=_{L}0$. Therefore, (3.6) is equivalent to

On the other hand, we recall that the Robinson-Colombeau ring  is already ordered by $\left\{ a_{n}\right\} _{n}=[a_{n\varepsilon }]_{\text {u}}\le [b_{n\varepsilon }]_{\text {u}}=\left\{ b_{n}\right\} _{n}$ if3.7We also recall that  through the embedding $x\mapsto [(x_{\varepsilon })_{n\varepsilon }]$. Thereby, a relation of the form $x\le [a_{n\varepsilon }]_{\text {u}}$ yields

$$\begin{aligned} \forall ^{0}\varepsilon \,\forall n\in \mathbb {N}:\ x_{\varepsilon }\le a_{n\varepsilon } \end{aligned}$$

for some representative $x=[x_{\varepsilon }]$. Finally, [13, Lem. 2] for the ring  implies that $\{a_{n}\}_{n}<\{b_{n}\}_{n}$ if and only if

$$\begin{aligned} \exists q\in \mathbb {R}_{>0}\,\forall ^{0}\varepsilon \,\forall n\in \mathbb {N}:\ b_{n\varepsilon }-a_{n\varepsilon }\ge \hbox {d}^{q}. \end{aligned}$$

#### Theorem 21

We have the following properties:

(i) is an ordered -module.
(ii) If $(a_{n})_{n}\ge 0$, then  is increasing.
(iii) If $\sigma \ge \rho ^{*}$, and $[a_{n\varepsilon }]_{{u }}\le [b_{n\varepsilon }]_{{u }}$ in , then $[a_{n\varepsilon }]_{{s }}\le [b_{n\varepsilon }]_{{s }}$ in .

#### Proof

(i): The relation $\le$ on  is clearly reflexive and transitive. If $(a_{n})_{n}\le (b_{n})_{n}\le (a_{n})_{n}$, then for all *N*,  we have $\sum _{n=N}^{M}a_{n}=\sum _{n=N}^{M}b_{n}$ in . From Definition 5 of hypersum, this implies (2.9), i.e. that $(a_{n})_{n}=(b_{n})_{n}$ in .

(ii): Let *N*,  with $N\le M$, then $\sum _{n=0}^{M}a_{n}=\sum _{n=0}^{N}a_{n}+\sum _{n=N+1}^{M}a_{n}$ by Lemma 14, and $\sum _{n=N+1}^{M}a_{n}\ge 0$ because $(a_{n})_{n}\ge 0$ in .

(iii): Assume that the inequality in (3.7) holds for all $\varepsilon \le \varepsilon _{0}$ and for all $n\in \mathbb {N}$. Then, if $N=[N_{\varepsilon }]$, $M=[M_{\varepsilon }]$, $N_{\varepsilon }$, $M_{\varepsilon }\in \mathbb {N}$, we have $\sum _{n=N_{\varepsilon }}^{M_{\varepsilon }}a_{n\varepsilon }\le \sum _{n=N_{\varepsilon }}^{M\varepsilon }b_{n\varepsilon }+\sum _{n=N_{\varepsilon }}^{M\varepsilon }z_{n\varepsilon }$ for all $\varepsilon \le \varepsilon _{0}$. Since , for each $q\in \mathbb {N}$, for $\varepsilon$ small and for all $n\in \mathbb {N}$, we have $|z_{n\varepsilon }|\le \rho _{\varepsilon }^{q}$ and hence $\left| \sum _{n=N_{\varepsilon }}^{M_{\varepsilon }}z_{n\varepsilon }\right| \le \left| M_{\varepsilon }-N_{\varepsilon }\right| \rho _{\varepsilon }^{q}$. Thereby, from the assumption $\sigma \ge \rho ^{*}$, it follows that $\left( \sum _{n=N_{\varepsilon }}^{M_{\varepsilon }}z_{n\varepsilon }\right) \sim _{\rho }0$, which proves the conclusion. $\square$

The direct comparison test for hyperseries can now be stated as follows.

#### Theorem 22

Let $\sigma$ and $\rho$ be arbitrary gauges, let  and  be hyperseries with $\left( a_{n}\right) _{n}$, $\left( b_{n}\right) _{n}\ge 0$ and such that

3.8 $$\begin{aligned} \exists N\in \mathbb {N}:\ (a_{n+N})_{n}\le (b_{n+N})_{n}. \end{aligned}$$

Then we have:

(i) If  is convergent then so is .
(ii) If  is divergent to $+\infty$, then so is .
(iii) If $\sigma \ge \rho ^{*}$ and $\{a_{n}\}_{n}{:=}[a_{n\varepsilon }]_{{u }}$, , then we have the same conclusions if we assume that

#### Proof

We first note that (3.8) can be simply written as $(\alpha _{n})_{n}\le (\beta _{n})_{n}$, where $\alpha _{n}{:=}a_{n+N}$ and $\beta _{n}{:=}b_{n+N}$. Thereby, Corollary 16 implies that, without loss of generality, we can assume $N=0$. To prove (i), let us consider the partial sums $A_{N}{:=}\sum _{i=0}^{N}a_{i}$ and $B_{N}{:=}\sum _{i=0}^{N}b_{i}$, . Since  is convergent, we have . The assumption $\left( a_{n}\right) _{n}\le \left( b_{n}\right) _{n}$ implies $A_{N}\le B_{N}$. The hypersequences ,  are increasing because $(a_{n})_{n}$, $(b_{n})_{n}\ge 0$ (Theorem 21.(ii)) and hence  decreases to zero because of the convergence assumption. Now, for all *N*, , $M\ge N$, we have

3.9 $$\begin{aligned} A_{N}\le A_{M}=\sum _{n=0}^{M}a_{n}=\sum _{n=0}^{N}a_{n}+\sum _{n=N+1}^{M}a_{n}\le A_{N}+\sum _{n=N+1}^{M}b_{n}=A_{N}+(B-B_{N}).\nonumber \\ \end{aligned}$$

Thereby, given *m*, $n\ge N$, applying (3.9) with *m*, *n* instead of *M*, we get that both $A_{n}$, $A_{m}$ belong to the interval $[A_{N},A_{N}+(B-B_{N})]$, whose length $B-B_{N}$ decreases to zero as  goes to infinity. This shows that  is a Cauchy hypersequence, and therefore  converges.

The proof of (ii) follows directly from the inequality $A_{N}\le B_{N}$ for each . The proof of (iii) follows from Lemma 21.(iii) and from Lemma 7.(iv). $\square$

Note that the two cases (i) and (ii) do not cover the case where the non-decreasing hypersums $N\mapsto \sum _{n=0}^{N}a_{n}$ are bounded but anyway do not converge because it does not exists the supremum of their values (see [13]).

#### Example 23

Let  be a finite number so that $x\le M\in \mathbb {N}_{>0}$ for some *M*. Assume that $x_{\varepsilon }\le M$ for all $\varepsilon \le \varepsilon _{0}$. For these $\varepsilon$ we have $\frac{n+x_{\varepsilon }}{n^{3}}\le \frac{2}{n^{2}}$ for all $n\in \mathbb {N}_{\ge M}$. This shows that $\left\{ \frac{n+M+x}{(n+M)^{3}}\right\} _{n}\le \left\{ \frac{2}{(n+M)^{2}}\right\} _{n}$ and we can hence apply Theorem 22.

The limit comparison test is the next

#### Theorem 24

Let $\sigma$ and $\rho$ be arbitrary gauges, let  and  be hyperseries, with $\left( a_{n}\right) _{n}\ge 0$. Assume that3.10Then we have:

(i) Either both hyperseries ,  converge or diverge to $+\infty$.
(ii) If $\sigma \ge \rho ^{*}$, $\left\{ a_{n}\right\} _{n}=[a_{n\varepsilon }]_{{u }}$, , $[b_{n\varepsilon }]_{{u }}>0$ and  then the same conclusion as in (i) holds.

#### Proof

As in the previous proof, without loss of generality, we can assume $N=0$. Now, if  diverges to $+\infty$, then so does  because $m>0$. Since $\left( mb_{n}\right) _{n}<\left( a_{n}\right) _{n}$, by the direct comparison test also the hyperseries  diverges to $+\infty$. Likewise, if the hyperseries  converges then so does . Since $\left( a_{n}\right) _{n}<\left( Mb_{n}\right) _{n}$, by the direct comparison test also the hyperseries  converges. Property (ii) can be proved as in the previous theorem. $\square$

Note that if *M*, $a_{n}$, $b_{n}\in \mathbb {R}$, then the condition $\left\{ \frac{a_{n}}{b_{n}}\right\} _{n}\le M$ is equivalent to $\limsup _{n\rightarrow +\infty }\frac{a_{n}}{b_{n}}\le M$. Analogously, $m\le \left\{ \frac{a_{n}}{b_{n}}\right\} _{n}$ is equivalent to $\liminf _{n\rightarrow +\infty }\frac{a_{n}}{b_{n}}\ge m$. This shows that our formulation of the limit comparison test faithfully generalizes the classical version.

Both the root and the ratio tests are better formulated in , because their proofs require term-by-term operations (such as e.g. that $|a_{n}|^{1/n}\le L$ implies $|a_{n}|\le L^{n}$), so that the order relation defined in Definition (20) seems too weak for these aims; see also the comment below the proof of the root test.

### Root test

#### Theorem 25

Let $\sigma \ge \rho ^{*}$ and let  (so that also ). Assume that3.11Then

(i) If $L<1$, then the hyperseries  converges absolutely.
(ii) If $J\subseteq _{0}I$ and $L>_{J}1$, then  and hence the hyperseries  diverges.
(iii) If $L\not =_{\mathrm{s}}1$, then the hyperseries  converges if and only if $L<1$.

#### Proof

(i): Our assumption $\left\{ |a_{n}|^{1/n}\right\} _{n}\le L$ entails $\left\{ |a_{n}|\right\} _{n}<\left( L^{n}\right) _{n}$. Since  is convergent because $0\le L<1$, by the direct comparison test, the series  is also convergent.

(ii): Now, assume that $L_{\varepsilon }>1$ for $\varepsilon \in J\subseteq _{0}I$ and let us work directly in the ring . Proceeding as above, we have $\left\{ |a_{n}|\right\} _{n}\ge _{J}\left( L^{n}\right) _{n}$, and thereby the last claim follows because of $L>_{J}1$.

(iii): Assume that $L\not =_{\mathrm{s}}1$, that  converges but $L>_{\mathrm{s}}1$, then (ii) would yield that  diverges for some $J\subseteq _{0}I$ and this contradicts the convergence assumption. Therefore, we have $L\not =_{\mathrm{s}}1$ and $L\not >_{\mathrm{s}}1$, and hence [13, Lem. 6.(v)] gives $L\not \ge _{\mathrm{s}}1$. Thereby, [13, Lem. 5.(ii)] finally implies $L<1$. $\square$

Actually, we can also simply reformulate the root test in  by trivially asking that $\left( \left| a_{n}\right| \right) _{n}\le \left( L^{n}\right) _{n}$, but that would simply recall a non-meaningful consequence of the direct comparison test Theorem 22.

### Ratio test

Proceeding as in the previous proof, i.e. by generalizing the classical proof for series of real numbers, we also have the following

#### Theorem 26

Let $\sigma \ge \rho ^{*}$ and let , with $\left\{ |a_{n}|\right\} _{n}>0$. Assume that for some $k\in \mathbb {N}$ we haveThen

(i) If $L<1$, the hyperseries  converges.
(ii) If $J\subseteq _{0}I$, $L>_{J}1$, then  and hence the hyperseries  diverges.
(iii) If $L\not =_{\mathrm{s}}1$, then the hyperseries  converges if and only if $L<1$.

As in the classical case, the convergent *p*-hyperseries  and the divergent hyperseries  shows that the ratio and the root tests fails if $L=1$.

#### Example 27

If  is a finite invertible number, then using the ratio test and proceeding as in Example 23, we can prove that  converges if $\sigma \ge \rho ^{*}$.

### Alternating series test

Also in the proof of this test we need a term-by-term comparison (see (3.14) below).

#### Theorem 28

Let $\sigma \ge \rho ^{*}$ and let . Assume that we have

3.12 $$\begin{aligned}&\left\{ a_{n+1}\right\} _{n}\le \left\{ a_{n}\right\} _{n} \end{aligned}$$

3.13Then the alternating hyperseries  converges.

#### Proof

By (3.12), assume that

3.14 $$\begin{aligned} a_{n+1,\varepsilon }\le a_{n\varepsilon }\quad \forall n\in \mathbb {N}\,\forall \varepsilon \le \varepsilon _{0} \end{aligned}$$

holds for all $n\in \mathbb {N}$ and all $\varepsilon \le \varepsilon _{0}$. Take $N=[N_{\varepsilon }]$, , where for $\varepsilon \le \varepsilon _{0}$ we have $N_{\varepsilon }$, $M_{\varepsilon }\in \mathbb {N}$. If $M_{\varepsilon }$ is odd and $M_{\varepsilon }\le N_{\varepsilon }$, we can estimate the difference $S_{N_{\varepsilon }}-S_{M_{\varepsilon }}$ as:

3.15 $$\begin{aligned} \begin{aligned}S_{N_{\varepsilon }}-S_{M_{\varepsilon }}&=\sum _{n=0}^{N_{\varepsilon }}(-1)^{n}a_{n}-\sum _{n=0}^{M_{\varepsilon }}(-1)^{n}a_{k}=\sum _{n=M_{\varepsilon }+1}^{N_{\varepsilon }}(-1)^{n}a_{n}\\&=a_{M_{\varepsilon }+1}-a_{M_{\varepsilon }+2}+a_{M_{\varepsilon }+3}+\cdots +a_{N_{\varepsilon }}\\&={\displaystyle a_{M_{\varepsilon }+1}-(a_{M_{\varepsilon }+2}-a_{M_{\varepsilon }+3})-(a_{M_{\varepsilon }+4}-a_{M_{\varepsilon }+5})-\cdots -a_{N_{\varepsilon }}}\\&\le a_{M_{\varepsilon }+1}\le a_{M_{\varepsilon }}, \end{aligned} \end{aligned}$$

where we used (3.14). If $M_{\varepsilon }$ is even and $M_{\varepsilon }\le N_{\varepsilon }$, a similar argument shows that $S_{N_{\varepsilon }}-S_{M_{\varepsilon }}\ge -a_{M_{\varepsilon }}$. If $M_{\varepsilon }>N_{\varepsilon }$, it suffices to revert the role of $M_{\varepsilon }$ and $N_{\varepsilon }$ in (3.15). This proves that $\min (-a_{M},-a_{N})\le S_{N}-S_{M}\le \max (a_{M},a_{N})$. The final claim now follows from (3.13) and Cauchy criterion [13, Thm. 44]. $\square$

#### Example 29

Using the previous Theorem 28, we can prove, e.g., that the hyperseries  is convergent.

### Integral test

The possibility to prove the integral test for hyperseries is constrained by the existence of a notion of generalized function that can be defined on an unbounded interval, e.g. of the form . This is not possible for an arbitrary Colombeau generalized function, which are defined only on finite (i.e. compactly supported) points, i.e. on domains of the form $\widetilde{\Omega }_{c}$ (see e.g. [9]). Moreover, we would also need a notion of improper integral extended on $[0,+\infty )$. This notion clearly needs to have good relations with the notion of hyperlimit and with the definite integral over the interval [0, *N*], where  (see Definition 32 below). This further underscores drawbacks of Colombeau’s theory, where this notion of integral (if *N* is an infinite number) is not defined, see e.g. [1].

The theory of generalized smooth functions overcomes these difficulties, see e.g. [7, 8]. Here, we only recall the equivalent definition. In the following, $\sigma$, $\rho$ always denote arbitrary gauges.

#### Definition 30

Let  and . We say that $f:X\longrightarrow Y$ is a $\rho$-moderate *generalized smooth function* (GSF), and we write , if

(i) $f:X\longrightarrow Y$ is a set-theoretical function.
(ii) There exists a net $(f_{\varepsilon })\in {\mathcal {C}}^{\infty }(\mathbb {R}^{n},\mathbb {R}^{d})^{(0,1]}$ such that for all $[x_{\varepsilon }]\in X$:
  - $$f(x)=[f_{\varepsilon }(x_{\varepsilon })]$$
  - $$\forall \alpha \in \mathbb {N}^{n}:\ (\partial ^{\alpha }f_{\varepsilon }(x_{\varepsilon }))\text { is }\rho -\text {moderate}$$.

For generalized smooth functions lots of results hold: closure with respect to composition, embedding of Schwartz’s distributions, differential calculus, one-dimensional integral calculus using primitives, classical theorems (intermediate value, mean value, Taylor, extreme value, inverse and implicit function), multidimensional integration, Banach fixed point theorem, a Picard-Lindelöf theorem for both ODE and PDE, several results of calculus of variations, etc.

In particular, we have the following

#### Theorem 31

Let *a*, *b*, , with $a<b$ and $c\in [a,b]\subseteq U$. Let  be a generalized smooth function. Then, there exists one and only one generalized smooth function  such that $F(c)=0$ and $F'(x)=f(x)$ for all $x\in [a,b]$. Moreover, if *f* is defined by the net $f_{\varepsilon }\in {\mathcal {C}}^{\infty }(\mathbb {R},\mathbb {R})$ and $c=[c_{\varepsilon }]$, then

3.16 $$\begin{aligned} F(x)=\left[ \int _{c_{\varepsilon }}^{x_{\varepsilon }}f_{\varepsilon }(s)\,\hbox {d}\right] \end{aligned}$$

for all $$x=[x_{\varepsilon }]\in [a,b]$$.

We can hence define $\int _{c}^{x}f(t)\,\hbox {d}{:=}F(x)$ for all $x\in [a,b]$ (note explicitly that *a*, *b* can also be infinite generalized numbers). For this notion of integral we have all the usual elementary property, monotonicity and integration by substitution included.

For the integral test for hyperseries, we finally need the following

#### Definition 32

Let , then we say

#### Theorem 33

Let  be a GSF such that

3.17 $$\begin{aligned} \forall ^{0}\varepsilon \,\forall n&\in \mathbb {N}\,\forall x\in [n,n+1)_{\mathbb {R}}:\ f_{\varepsilon }(x)\le f_{\varepsilon }(n) \end{aligned}$$

3.18 $$\begin{aligned} \forall ^{0}\varepsilon \,\forall n&\in \mathbb {N}_{>0}\,\forall x\in [n-1,n)_{\mathbb {R}}:\ f_{\varepsilon }(n)\le f_{\varepsilon }(x) \end{aligned}$$

then  and

(i) The hyperseries  converges if $\exists \int _{0}^{+\infty }f(x)\,\hbox {d}$ and .
(ii) The hyperseries  diverges to $+\infty$ if $\int _{0}^{+\infty }f(x)\,\hbox {d}=+\infty$.

#### Proof

From (3.16), for all *N*,  we have

$$\begin{aligned} \int _{N}^{M+1}f(x)\,\hbox {d}=\left[ \int _{N_{\varepsilon }}^{M_{\varepsilon }+1}f_{\varepsilon }(x)dx\right] . \end{aligned}$$

Without loss of generality, we can assume $N_{\varepsilon }$, $M_{\varepsilon }\in \mathbb {N}$ for all $\varepsilon$. Thereby

$$\begin{aligned} \int _{N}^{M+1}f(x)dx&=\left[ \sum _{n=N_{\varepsilon }}^{M_{\varepsilon }}\int _{n}^{n+1}f_{\varepsilon }(x)\,\hbox {d}\right] \le \\&=\left[ \sum _{n=N_{\varepsilon }}^{M_{\varepsilon }}\int _{n}^{n+1}f_{\varepsilon }(n)\,\hbox {d}\right] =\sum _{n=N}^{M}f(n), \end{aligned}$$

where we used Definition 5 of hypersum and (3.17). On the other hand, since , using (3.18) we have

$$\begin{aligned} \sum _{n=N}^{M}f(n)&=f(N)+\sum _{n=N+1}^{M}\int _{n-1}^{n}f(n)\,\hbox {d}=f(N)+\left[ \sum _{n=N_{\varepsilon }+1}^{M_{\varepsilon }}\int _{n-1}^{n}f_{\varepsilon }(n)\,\hbox {d}\right] \\&\le f(N)+\left[ \sum _{n=N_{\varepsilon }+1}^{M_{\varepsilon }}\int _{n-1}^{n}f_{\varepsilon }(x)\,\hbox {d}\right] =f(N)+\int _{N}^{M}f(x)\,\hbox {d}, \end{aligned}$$

where we used again Definition 5 of hypersum and (3.16). Combining these two inequalities yields

3.19 $$\begin{aligned} \int _{N}^{M+1}f(x)\,\hbox {d}\le \sum _{n=N}^{M}f(n)\le f(N)+\int _{N}^{M}f(x)\,\hbox {d}. \end{aligned}$$

These inequalities show that . Now, we consider that $\sum _{n=0}^{M}f(n)-\sum _{n=0}^{N}f(n)=\sum _{n=N}^{M}f(n)$ for $M\ge N$, and

$$\begin{aligned} \int _{N}^{M}f(x)\,\hbox {d}=-\int _{0}^{N}f(x)\,\hbox {d}+\int _{0}^{M}f(x)\,\hbox {d}. \end{aligned}$$

Therefore, if $\exists \int _{0}^{+\infty }f(x)\,\hbox {d}$ and , letting *N*, $M\rightarrow +\infty$ in (3.19) proves that our hyperseries satisfies the Cauchy criterion. If $\int _{0}^{+\infty }f(x)\,\hbox {d}=+\infty$, then setting $N=0$ in the first inequality of (3.19) and $M\rightarrow +\infty$ we get the second conclusion. $\square$

Note that, generalizing by contradiction the classical proof, we only haveand not $L=0$ like in classical case.

As usual, from the integral test we can also deduce the *p*-series test:

#### Corollary 34

If , then the *p*-hyperseries converges.

#### Proof

Since $p=[p_{\varepsilon }]>1$, we can assume that $p_{\varepsilon }>1$ for all $\varepsilon \le \varepsilon _{0}$. For these $\varepsilon$, if $x\in [n,n+1)_{\mathbb {R}}$, we also have $\frac{1}{x^{p_{\varepsilon }}}\le \frac{1}{n^{p_{\varepsilon }}}$, which proves (3.17). Similarly, we can prove (3.18). Note that the function $f(x)=\frac{1}{x^{p}}$ for all  is $\rho$-moderate because $x\ge 1$ and $p>1$, so that $\left| f(x)\right| \le 1$. Finally, like in the classical case, we have  because $p>1$. $\square$

Note, however, that our proofs in Sect. 3.1 are independent from the notion of generalized smooth function.

## Cauchy product of hyperseries

We recall that, on the basis of Theorem 3, the operations of sum and product by a number in  are the sole operations defined in . We now consider the classical Cauchy product:

### Definition 35

The *Cauchy product of two sequences for hypersums* $\left( a_{n}\right) _{n}$,  is defined as

$$\begin{aligned} \left( a_{n}\right) _{n}\star \left( b_{n}\right) _{n}{:=}\left( \sum _{k=0}^{n}a_{k}b_{n-k}\right) _{n}. \end{aligned}$$

The *Cauchy product of two hyperseries* is the hyperlimit of the hypersums with general term $\left( a_{n}\right) _{n}\star \left( b_{n}\right) _{n}$, assuming that it exists:A similar *product of sequences* can be defined in  with

$$\begin{aligned} \left\{ a_{n}\right\} _{n}\star \left\{ b_{n}\right\} _{n}{:=}\left\{ \sum _{k=0}^{n}a_{k}b_{n-k}\right\} _{n}. \end{aligned}$$

### Theorem 36

The module  is a ring with respect to the Cauchy product of sequences. The ring  is also a ring with respect to the Cauchy product of sequences.

### Proof

Let $\left( a_{n}\right) _{n}$, , $(a_{n\varepsilon })_{n,\varepsilon }\sim _{\sigma \rho }(\bar{a}_{n\varepsilon })_{n,\varepsilon }$, $(b_{n\varepsilon })_{n,\varepsilon }\sim _{\sigma \rho }(\bar{b}_{n\varepsilon })_{n,\varepsilon }$. We first prove that the Cauchy product $\left( a_{n}\right) _{n}\star \left( b_{n}\right) _{n}$ is well-defined in . For simplicity, for *n*, , set $A_{nm}{:=}\sum _{k=n}^{m}a_{k}$, $B_{nm}{:=}\sum _{k=n}^{m}b_{k}$ and similar notations $\bar{A}_{nm}$, $\bar{B}_{nm}$ using the other representatives. Then

$$\begin{aligned} \sum _{k=n}^{m}a_{k}b_{n-k}-\sum _{k=n}^{m}\bar{a}_{k}\bar{b}_{n-k}=\left( A_{nm}-\bar{A}_{nm}\right) B_{nm}+\bar{A}_{nm}\left( B_{nm}-\bar{B}_{nm}\right) . \end{aligned}$$

Now, $A_{nm}-\bar{A}_{nm}$ and $B_{nm}-\bar{B}_{nm}$ are $\rho$-negligible by Definition (2.9) of $\sim _{\sigma \rho }$, and $B_{nm}$, $\bar{A}_{nm}$ are $\rho$-moderate because of Lemma 4. This proves that the Cauchy product $\star$ in  is well-defined. Similarly, we can proceed with . The ring properties now follow from the same properties of the ring . $\square$

It is well-known that for each $n\in \mathbb {N}$, we have $\left( a_{n}\right) _{n}\star \left( b_{n}\right) _{n}=\left( \sum _{i=0}^{n}a_{i}\right) \cdot \left( \sum _{j=0}^{n}b_{j}\right) =\sum _{i=0}^{n}\sum _{j=0}^{n}a_{i}b_{j}=:c_{n}$, so  by Theorem 36. Moreover, if  and  converge, taking the hyperlimit of the product of the two partial sums we obtainbut, in general, this is different fromIn other words, if the Cauchy product of these hyperseries exists, in general we haveThe classical Mertens’ theorem also holds for hyperseries:

### Theorem 37

Assume that the hyperseries  converges to *A* and  converges to *B*. Assume that the former hyperseries converges absolutely. Then their Cauchy product converges to *AB*.

We first need the following

### Lemma 38

If $(b_{n})_{n}$ converges to *B*, then4.1

### Proof

Set  for all . The assumption of convergence yields4.2Set  and let . We use dichotomy law as in [13, Lem. 7.(ii)], i.e. we consider $L\subseteq _{0}I$ and two cases $N\ge _{L}N_{1}$ or $N\le _{L}N_{1}$. We claim that $\left| B_{N_{\varepsilon }}-B_{\varepsilon }\right| \le K_{\varepsilon }$ for all $\varepsilon \in L$ sufficiently small, where $N_{\varepsilon }{:=}\mathrm{ni}{(N)}_{\varepsilon }$, $N_{1\varepsilon }{:=}\mathrm{ni}{(N_{1})}_{\varepsilon }$, , $B_{N_{\varepsilon }}{:=}\sum _{k=0}^{N_{\varepsilon }}b_{k\varepsilon }$, $B=[B_{\varepsilon }]$ and $K_{\varepsilon }{:=}1+\sum _{k=0}^{N_{1\varepsilon }}\left| b_{k\varepsilon }\right|$ (hence $K=[K_{\varepsilon }]$). We also note that, in general, *P*, , $P\le Q$ implies $\mathrm{ni}{(P)}_{\varepsilon }\le \mathrm{ni}{(Q)}_{\varepsilon }$ for $\varepsilon$ small. In the first case $N\ge _{L}N_{1}$, we get $N_{\varepsilon }\ge N_{1\varepsilon }$ for $\varepsilon$ small. Set $M_{\varepsilon }{:=}N_{\varepsilon }$ if $\varepsilon \in L$ and $M_{\varepsilon }{:=}N_{1\varepsilon }$ otherwise, so that  and from (4.2) we obtain $\left| B_{M_{\varepsilon }}-B_{\varepsilon }\right| <1$ for $\varepsilon$ small and, in particular

$$\begin{aligned} \forall ^{0}\varepsilon \in L:\ \left| B_{N_{\varepsilon }}-B_{\varepsilon }\right| <1\le K_{\varepsilon }. \end{aligned}$$

We claim the same conclusion also in the second case $N\le _{L}N_{1}$, i.e.

4.3 $$\begin{aligned} \forall ^{0}\varepsilon \in L:\ N_{\varepsilon }\le N_{1\varepsilon }. \end{aligned}$$

In fact, we have $B_{N}=B_{N_{1}\vee N}-\sum _{k=N+1}^{N_{1}\vee N}b_{k}$ and hence

4.4 $$\begin{aligned} \left| B_{N}-B\right|&\le \left| B_{N_{1}\vee N}-B\right| +\sum _{k=N+1}^{N_{1}\vee N}|b_{k}|<1 \nonumber \\&\quad +\sum _{k=0}^{N_{1}\vee N}|b_{k}| \forall ^{0}\varepsilon :\ \left| B_{N_{\varepsilon }}-B_{\varepsilon }\right| \le 1+\sum _{k=0}^{N_{1\varepsilon }\vee N_{\varepsilon }}|b_{k\varepsilon }|. \end{aligned}$$

Inequalities (4.3) and (4.4) prove the claim also in the second case. Therefore, the dichotomy law yields $|B_{N}-B|\le K$ and *K* does not depend on *N*. $\square$

### Proof of Theorem 37

Define $A_{n}{:=}\sum _{i=0}^{n}a_{i}$, $B_{n}{:=}\sum _{i=0}^{n}b_{i}$ and $C_{n}{:=}\sum _{i=0}^{n}c_{i}$ for , and with $c_{i}{:=}\sum _{k=0}^{i}a_{k}b_{i-k}$ for $i\in \mathbb {N}$. Then $C_{n}=\sum _{i=0}^{n}a_{n-i}B_{i}$ because the same equality holds $\varepsilon$-wise for any representatives, and hence $C_{n}=\sum _{i=0}^{n}a_{n-i}(B_{i}-B)+A_{n}B$. The idea is to estimate

4.5 $$\begin{aligned} \left| C_{n}-AB\right|&=\left| \sum _{i=0}^{n}a_{n-i}(B_{i}-B)+(A_{n}-A)B\right| \nonumber \\&\le \sum _{i=0}^{N-1}|a_{n-i}||B_{i}-B|+\sum _{i=N}^{n}|a_{n-i}||B_{i}-B|+|A_{n}-A||B|, \end{aligned}$$

and to use our assumption (4.1) for the first summand. We start from the second summand in (4.5): Since  converges absolutely to some $\bar{A}$ and $B_{n}$ converges to *B*, for all $q\in \mathbb {N}$ we can find  such that

$$\begin{aligned} \left| B_{n}-B\right| \le \frac{\hbox {d}^{q}}{\bar{A}+1} \end{aligned}$$

for all $n\ge N$. Therefore, for the same $n\ge N$

$$\begin{aligned} \sum _{i=N}^{n}|a_{n-i}||B_{i}-B|\le \hbox {d}^{q}. \end{aligned}$$

First summand in (4.5): Since  converges,  must converge to zero. Hence for some  and for all $n\ge M$ and because of Lemma 38, we have

4.6 $$\begin{aligned} \left| a_{n}\right| \le \frac{\hbox {d}^{q}}{N(K+1)}\le \frac{\hbox {d}^{q}}{N\left( \left| B_{i}-B\right| +1\right) }\quad \forall i\le N-1 \end{aligned}$$

In particular, if $n\ge M+N$ and $i\le N-1$, then $n-i\ge n-N+1>M$ and we can apply (4.6) with $n-i$ instead of *n* obtaining

$$\begin{aligned} \sum _{i=0}^{N-1}|a_{n-i}||B_{i}-B|\le \frac{\hbox {d}^{q}N}{N}. \end{aligned}$$

Finally, since $A_{n}$ converges to *A*, for some  and for all $n\ge L$, we have

$$\begin{aligned} \left| A_{n}-A\right| \le \frac{\hbox {d}^{q}}{|B|+1}. \end{aligned}$$

Then, for all $n\ge \max \left( L,M+N\right)$ using the aforementioned estimates, (4.5) yields $\left| C_{n}-AB\right| \le 3\hbox {d}^{q}$. $\square$

## Conclusions

One of the main goals of the present paper is to show that when dealing with non-Archimedean Cauchy complete rings, it can be worth to consider summations over infinite natural numbers instead of classical series indexed by $n\in \mathbb {N}$. As we proved for the Robinson-Colombeau ring , this allows us to extend numerous classical results which do not hold using classical series in a non-Archimedean framework. We tried to motivate in a clear way why we have to consider two gauges $\sigma$ and $\rho$ and, for each result, what relationships we have to consider among them. To help the reader to summarize the condition on the gauges, we can use the following table

**Table:** 

	$\sigma$, $\rho$ arbitrary
	$$\sigma \ge \rho ^{*}$$
Divergent hypersums	and $\sigma \ge \rho ^{*}$
Geometric hyperseries	$\sigma \ge \rho ^{*}$ or $\sigma \le \rho ^{*}$
Taylor series exponential	$\sigma \ge \rho ^{*}$ or $\sigma \le \rho ^{*}$
$\varepsilon$-wise convergence	$\exists \sigma \le \rho ^{*}$ or $\sigma =\rho$
*p*-series	$\sigma$, $\rho$ arbitrary
Direct comparison test	$\sigma$, $\rho$ arbitrary
Limit comparison test	$\sigma$, $\rho$ arbitrary
Root test	and $\sigma \ge \rho ^{*}$
Ratio test	and $\sigma \ge \rho ^{*}$
Alternating series test	and $\sigma \ge \rho ^{*}$
Integral test	$\sigma$, $\rho$ arbitrary

Therefore, a possible summary could be: for meaningful examples of convergent series, for all convergence tests, one can assume $\sigma \ge \rho ^{*}$ and use both  or . For $\varepsilon$-wise convergence, to have larger domains (see Example 11.10)), one can assume $\sigma \le \rho ^{*}$ and hence use . For divergent hypersums, one must assume $\sigma \ge \rho ^{*}$ and work in .

Since non-Archimedean rings are not Dedekind-complete, we have been forced to substitute the least-upper-bound property using the weaker Cauchy completeness. We think that several of the proofs we presented here can be generalized to other non-Archimedean Cauchy complete settings.

Clearly, the present work opens the actual possibility to start the study of hyper-power series, (real or complex) hyper-analytic generalized smooth functions and sigma additivity of multidimensional integration of generalized smooth functions (see [8]).

We also finally mention that the embedding of Schwartz’s distributions can be realized by regularization with an *entire* Colombeau mollifier (see e.g. [9]). We can hence conjecture that this embedding yields a hyper-analytic generalized smooth functions (but note that the hyper-power series of classical non-analytic smooth functions with a flat point would converge to zero under any invertible infinitesimal). This conjecture would open the possibility to extend the Cauchy-Kowalevski theorem to all Schwartz’s distributions.
